# How do environmental and cultural factors shape red tourism behavioral intentions: a moderated mediation model

**DOI:** 10.3389/fpsyg.2025.1566533

**Published:** 2025-07-09

**Authors:** Cong Chen, Yinghui Lai, Chenjing Huo

**Affiliations:** ^1^School of Educational Sciences, Hunan Institute of Science and Technology, Yueyang, China; ^2^School of Educational Science, Hunan Normal University, Changsha, China; ^3^School of Foreign Studies, Hebei Normal University, Shijiazhuang, China

**Keywords:** red tourism experience, environmental restorativeness perception, post-visit behavioral intentions, cultural identity, stimulus-organism-response theory

## Abstract

**Introduction:**

This study investigates how environmental restorativeness perception and cultural identity shape the relationship between red tourism experience and post-visit behavioral intentions.

**Methods:**

A structured questionnaire was administered to 1,195 tourists at two iconic red tourism destinations in China, Xibaipo and Shaoshan. Key constructs, including red tourism experience, environmental restorativeness perception, cultural identity, and post-visit behavioral intentions, were assessed using validated multi-item scales. After controlling for gender, age, and education as covariates, latent moderated structural equation modeling (LMS) was employed to analyze mediation and moderation effects.

**Results:**

The results indicate that environmental restorativeness perception significantly mediates the influence of red tourism experience on post-visit behavioral intentions. Cultural identity significantly moderates the first stage of this mediation pathway, such that higher cultural identity strengthens the positive effect of red tourism experience on perceived restorativeness.

**Discussion:**

These findings suggest that red tourism environments can restore attention and reduce stress while simultaneously reinforcing collective identity, thus promoting loyalty behaviors. The study contributes to environmental psychology and heritage tourism by highlighting the dual pathway through which ideological landscapes foster both emotional recovery and socio-cultural engagement.

## 1 Introduction

Red tourism is a distinctive form of communist heritage tourism (Qian et al., 2024), and it encompasses thematic activities that commemorate and reflect on the revolutionary history, deeds, and spirit at memorial sites associated with the Communist Party of China’s leadership during the revolutionary period (Sun and Lv, 2025). Although the red tourism market has reached a trillion-yuan scale (Wang, 2024), it faces several challenges: limited depth and breadth of tourist experiences, inadequate integration of natural and cultural resources, and insufficient exploration of the intrinsic value of red culture (Calderon-Fajardo, 2023). These limitations result in low tourist loyalty and revisit rates (Dai et al., 2023). How can red tourism experiences be converted into long-term behavioral intentions? What psychological mechanisms underpin this transformation? Addressing these questions is crucial for enhancing the preservation of Chinese red cultural heritage and revitalizing the economies of revolutionary base areas. Moreover, it offers important insights for the global advancement of political heritage tourism.

Post-visit behavioral intentions are strategically vital for tourism branding and market expansion. Many tourism destinations report that repeat visitors account for over 50% of their total visitor base (Karakan and Birdir, 2023). Enhancing existing tourists’ positive post-visit behavioral intentions can significantly lower marginal acquisition costs compared to attracting new visitors (Guo and Huang, 2013). Although existing studies have explored the factors influencing post-visit behavioral intentions through traditional dimensions such as travel motivation, perceived value, and destination image (Karakan and Birdir, 2023; Guo and Huang, 2013), the focus on tourists’ subjectivity in red tourism remains insufficient under the evolving context of the experience economy (Xin et al., 2023). The psychological mechanisms driving the transformation of experiential elements into behavioral outcomes lack theoretical clarification.

Previous studies have increasingly focused on how the restorative properties of tourism destinations, defined as their capacity to restore visitors’ mental resources, influence post-visit behavioral intentions. Theoretical frameworks, such as attention restoration theory (ART) (Kaplan and Kaplan, 1989), have been employed to elucidate how restorative environmental qualities affect tourists’ cognitive, emotional, and behavioral responses. However, empirical findings in this area remain inconsistent. Some studies report that higher environmental restorativeness perception leads to greater satisfaction and loyalty (Zhu et al., 2025), whereas others find that even when environmental factors enhance visitor satisfaction, they do not necessarily translate into stronger revisit intentions (Fatmawati and Olga, 2023). Such inconsistencies suggest that contextual and individual factors may moderate the relationship between environmental restorativeness perception and post-visit behavioral intentions.

From a political heritage tourism perspective, heritage tourists typically exhibit characteristics such as high expenditure, extended stays, and strong cultural demands (Kaur and Kaur, 2020). Comparable forms of tourism, such as military, patriotic, and red tourism, share experiential and psychological commonalities: they activate collective memory and intense emotions while reinforcing ethnic and national identity (Çakar, 2020; Zhao and Timothy, 2015). Cross-cultural studies indicate that heritage tourism and cultural identification are closely interconnected, with diverse cultural identity backgrounds significantly influencing visitor behavior at political heritage sites (Bechtel, 2016). However, prior research has predominantly emphasized emotional responses to trauma landscapes (Oren et al., 2022), typically framed through a “trauma landscape–emotional resonance–responsible behavior” pathway. Few studies, however, have examined how healing landscapes facilitate post-visit behavioral intentions. Adopting a positive perspective, this study uses red tourism as a representative setting to examine environmental restorativeness perception and post-visit behavioral intentions within a broader theoretical context. It further explores how cultural identity moderates tourist experiences and behavioral outcomes in political heritage tourism contexts.

In summary, this study integrates the experience economy theory (Pine and Gilmore, 1999), stimulus–organism–response theory (SOR) (Mehrabian and Russell, 1974), and ART, while incorporating cultural identification and environmental restorativeness perception to address two core research questions.

First, how does red tourism experience shape post-visit behavioral intentions through environmental restorativeness perception? In the post-pandemic era, tourism demand increasingly reflects wellness-oriented preferences (Xue et al., 2023), with growing public awareness of and demand for the restorative potential of tourism environments (De Gregorio et al., 2023; Dong and Qu, 2023). Red tourism sites, often situated in former revolutionary regions with robust ecological conditions, their “fascinating” and “sacred” environmental qualities aligned with restorative environmental characteristics described in ART (Zhang and Li, 2024). However, most existing research emphasizes the political-educational role of red tourism (Liu et al., 2021), with limited attention to its physical and psychological restorative potential. This study examines Xibaipo and Shaoshan, two sites that integrate revolutionary heritage clusters with natural landscapes, achieving a dynamic balance between ideological symbolism and Eastern aesthetic values. The unique spatial and symbolic features of this revolutionary geography remain underexplored within the current SOR theoretical framework.

Second, how does cultural identification moderate the relationship among tourism experience, environmental restorativeness perception, and post-visit behavioral intentions? Cultural identification is often triggered by immersive experiences, such as historical reenactment and emotional resonance with heroic narratives, which in turn foster intentions to revisit, preserve, and advocate for the sites. However, limited research has examined how cultural identification dynamically forms within tourism settings (Fu and Luo, 2023), particularly in red tourism contexts. Addressing these issues contributes to the development of a politicized restorativeness framework, bridging theoretical gaps in understanding the psychological mechanisms of ideological landscapes.

## 2 Literature review and research hypotheses

### 2.1 The relationship between red tourism experience and post-visit behavioral intention

The tourism experience is central to tourism products (Dorcic, 2024; Zhang et al., 2018), highlighting key elements such as interaction, co-creation, embodiment, psychology, and spirituality within the experiential environment (Blumenthal and Jensen, 2019; Chen et al., 2020; Tresidder and Deakin, 2019). Post-visit behavioral intentions reflect tourists’ attitudes toward a destination, encompassing intentions to revisit and intentions to recommend (Chen and Tsai, 2007). Experience economy theory reveals that tourists in passive reception states primarily seek superficial satisfaction through entertainment and aesthetic perception. Conversely, when their subjectivity is activated, tourists can achieve cognitive transformation through educational internalization and situational escapism (Jiang et al., 2024; Wiyata et al., 2024). This value transformation process aligns with the SOR model: multidimensional stimuli (S) received by tourists in red tourism settings undergo organism conversion through cognitive evaluation and emotional resonance (O), ultimately driving the formation of post-visit behavioral intentions (R).

Tourism experience can directly or indirectly influence post-visit behavioral intentions (Barnes et al., 2016; Karakan and Birdir, 2023). Previous research has shown that memorable tourism experience positively influence the willingness to revisit (Petrick, 2004), while tourism experience quality positively affects place attachment (Zhang J. et al., 2024; Zhang R. et al., 2024). Social interactions during tourism affect post-visit behavioral intentions (Zhou G. et al., 2023; Zhou H. et al., 2023). Moreover, tourism experience influences revisit decisions via episodic future thinking (Zhao et al., 2023), and indirectly affects behavioral intentions through perceived value and satisfaction (Chen and Chen, 2010; Sun et al., 2019). The evidence suggests that tourism experience, such as performing arts, enjoyable learning, and emotional experiences, indirectly influence destination loyalty through destination image and satisfaction Specifically, red tourism studies show that the experiences affects tourists’ intentions to visit similar destinations via factors such as national identity, destination image, place attachment, and overall satisfaction (Zhou et al., 2022). Accordingly, we propose our first hypothesis:

*H1:* Red tourism experience has a significantly positive effect on post-visit behavioral intentions.

### 2.2 Mediating role of environmental restorativeness perception

Environmental restorativeness perception is one of the organismic variables closely linked to the external environment, reflecting an individual’s perceived recovery from negative states such as fatigue within a specific environment (Chen and Xi, 2018). The ART posits that restorative environments are energizing, clearing the mind, enhancing positive emotions, and enabling deep reflection, which in turn promote self-awareness and personal growth (Kaplan and Kaplan, 1989; Stragà et al., 2023). Among these, reflection embodies a profound perception of restoration. Since the COVID-19 pandemic, heightened awareness and demand for environmental restorative functions (De Gregorio et al., 2023; Dong and Qu, 2023) imply that environmental restorativeness perception might mediate the link between red tourism experience and subsequent behavioral intentions.

On the one hand, positive tourism experiences can enhance environmental restorativeness perception. Research has demonstrated that the visual and acoustic landscape features of natural destinations are fundamental to attention restoration (Qiu et al., 2021). Both the perception of soundscapes (Liu et al., 2020) and natural experiences (Tang et al., 2024) in rural tourism settings can promote restorative perception. The experiences of beach temperature and air quality also affect environmental restorativeness perception (Hipp and Ogunseitan, 2011). Positive experiences in various forms of tourism and destinations can effectively alleviate stress (Chen et al., 2016). Scenario experiments indicate that red tourism serves as a psychological and physiological “battery” or “charger” for tourists (Zhang and Li, 2024). Strong emotional resonance during red tourism can quickly evoke positive emotions and profound feelings, prompt profound reflection on real-life experiences (Gill et al., 2019), and ultimately promote cognitive and attitudinal changes (Liu et al., 2018).

On the other hand, environmental restorativeness perception has a positive effect on post-visit behavioral intentions. Wu et al. (2024) reported that environmental restorativeness perception significantly affects tourists’ place attachment to urban parks. A survey of Hangang Park in South Korea revealed that the coherence and compatibility dimensions of environmental restorativeness perception positively influence place attachment (Yoon and Kim, 2020). Chen and Xi (2018) and Huang et al. (2022) conducted research in natural scenic areas like Kanas and Nankun Mountain in Guangdong. They reported that tourists’ environmental restorativeness perception positively predicts their post-visit behavioral intentions. However, the two studies differed in their conclusions about the role of the subdimensions of this perception. The former holds that the fascination and compatibility dimensions of tourists’ environmental restorativeness perception directly and positively influence post-visit behavioral intentions, with the contribution of fascination being slightly greater than that of compatibility. The latter suggests that the novelty and escape dimensions are the primary drivers for generating post-visit behavioral intentions. Hence, we posit the second hypothesis:

*H2:* Environmental restorativeness perception mediates the relationship between red tourism experience and post-visit behavioral intentions.

### 2.3 Moderating role of cultural identity

Tourists’ cultural identity with the destination significantly drives their travel behavior. Strengthening cultural identity can shape tourists’ destination choices (Usunier, 2006). Cultural identification reflects the extent of alignment between an individual’s cognition, attitudes, and behaviors and those prevalent in a given culture (Ferguson et al., 2017). The person-environment fit theory posits that when an individual’s traits (e.g., personality, values, needs) align with environmental traits (e.g., social culture, physical environment), they are more prone to display positive attitudes and behaviors (White et al., 2022). Research indicates that a profound experience and favorable impressions of the destination culture can enhance tourists’ behavioral intentions (Coudounaris and Sthapit, 2017). In heritage tourism, aesthetic and interactive engagement fosters psychological experiences that enhance the understanding of cultural connotations, awaken cultural genes, and deepen cultural identification. When tourists identify strongly with a heritage site’s culture, they participate more actively in tourism activities, gain deeper appreciation of the cultural landscape, and consequently experience richer psychological outcomes. Tourists opt to revisit or recommend destinations because of the favorable experience induced by their cultural identity (Fu and Luo, 2023). Furthermore, cultural identification can inspire patriotism and national pride, even in suboptimal tourism experiences, motivating re-engagement and learning about culture (Wang, 2024). Therefore, an interaction between red tourism experience and cultural identity may influence the post-visit behavioral intentions.

Cultural identity represents the emotional bond between an individual and a specific culture, which may shape environmental preferences for tourist destinations, thereby forming and regulating environmental restorativeness perceptions (Wilkie and Clouston, 2015). Currently, research on the influence of tourism experience and cultural identity on environmental restorativeness perception is insufficient. Rural tourism studies suggest that cultural memory spaces enhance visitors’ environmental restorativeness perception via situational involvement (Chang and Li, 2022). The interaction of heritage tourism experiences and cultural identity augments the aesthetic perception of the tourism setting (Yang et al., 2022). Thus, travel experiences and cultural identity likely interact to shape environmental restorativeness perception.

Moreover, prior studies have focused on the combined impact of the tourism experience, memory, and satisfaction on behavioral intentions (Ali et al., 2016; Altunel and Erkut, 2015; Chen and Chen, 2010), yet few have integrated environmental restorativeness perception and cultural identity in this context. Research has revealed that tourism nostalgia and place attachment mediate the relationship between the rural soundscape experiences and the environmental restorativeness perception (Liu et al., 2020). Cultural attachment to restorative settings like temples can influence tourist loyalty (Xu and Hu, 2024). Differences in restorative qualities exist between natural and historical built environments at cultural heritage sites. The restorativeness of natural environment is characterized by traits like “escape,” whereas the historical built environment is defined by “fascination” (Scopelliti et al., 2019). This implies that tourists’ emotional connection to history and culture, alongside natural experiences, could jointly shape tourism decisions and behaviors. Consequently, this study hypothesizes the following:

*H3:* Cultural identity significantly moderates the mediation of “red tourism experience, environmental restorativeness perception, and post-visit behavioral intentions.”

**Figure 1:**
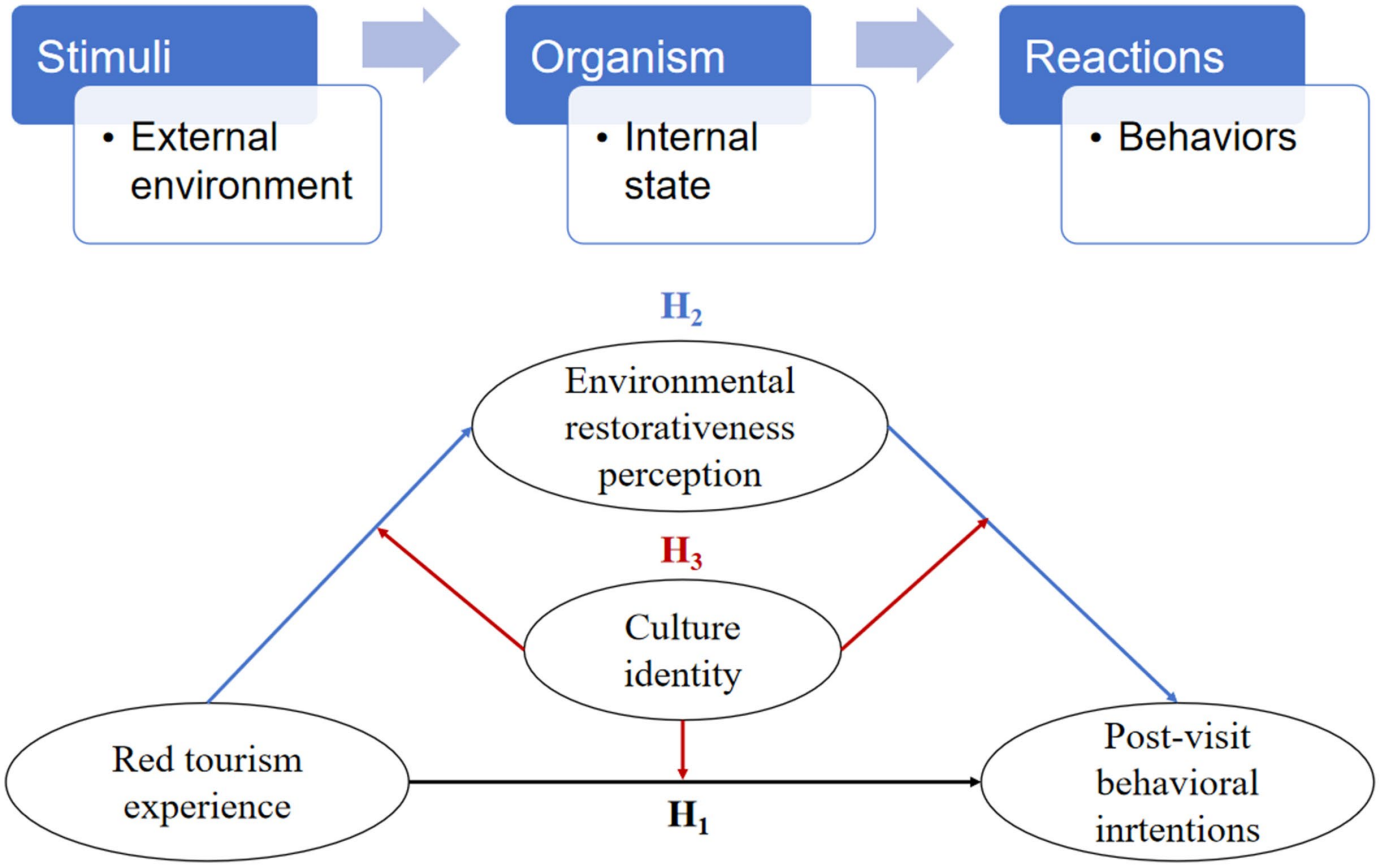
The hypothetical model.

In summary, drawing on the SOR theory and ART, the present study constructs a moderated mediation model (Figure 1) to examine the mediating role of environmental restorativeness perception between the red tourism experience and post-visit behavioral intentions, as well as the moderating role of cultural identity in this mediating process. Considering that age, gender, and education level may be related to post-visit behavioral intentions (Rather and Hollebeek, 2021), these variables are included as control variables in the model testing.

## 3 Methods

### 3.1 Research sites and participants

**Figure 2:**
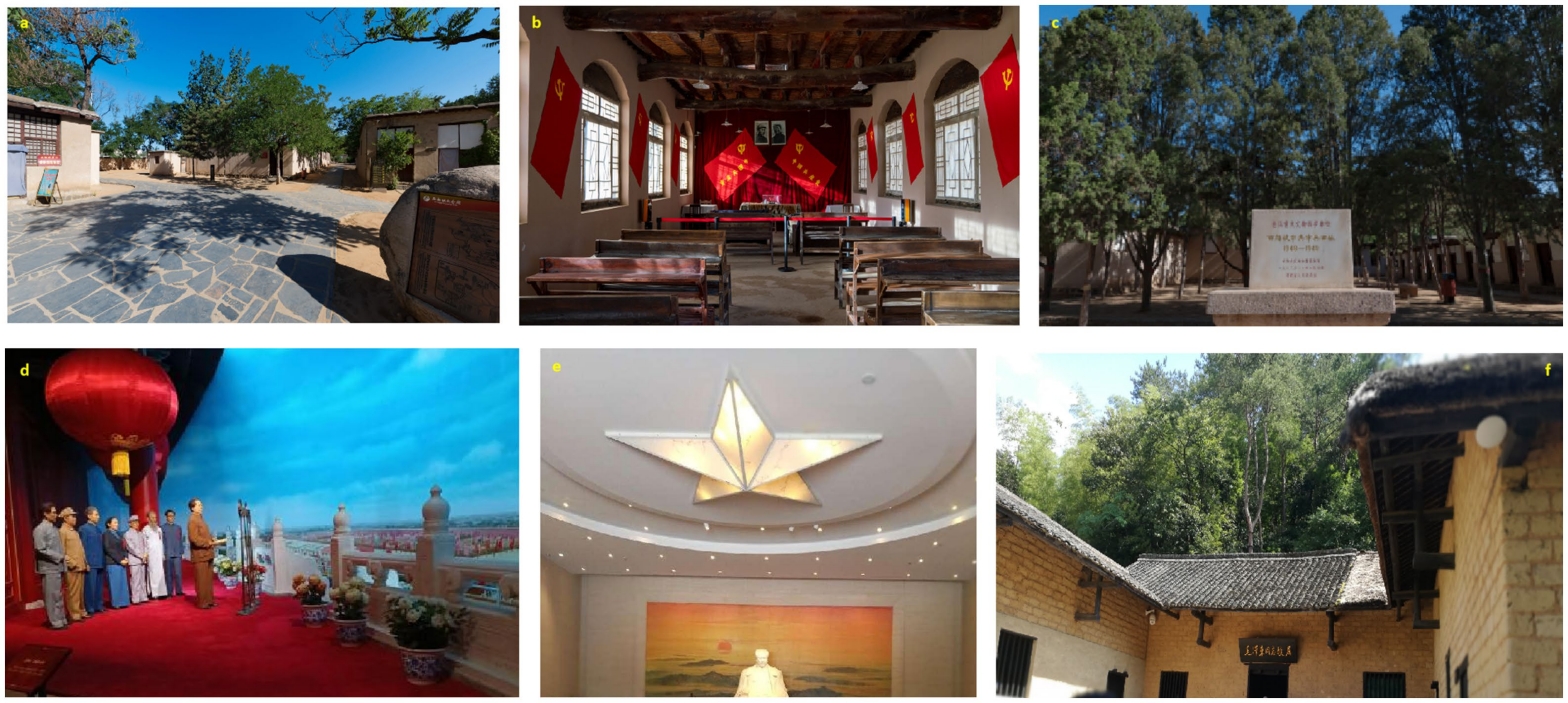
Landscape of the research sites. **a-c** denote red tourism sites in Xibaipo, and **d-f** denote red tourism sites in Shaoshan.

Xibaipo and Shaoshan were selected as the research sites because both are renowned red tourism destinations in China, attracting numerous visitors and possessing significant historical and cultural value, with extensive market influence and representativeness. The Xibaipo scenic area is a significant historical site for the Chinese Communist Party, renowned as the place “where the new China came from.” The Shaoshan scenic area is the birthplace of Chairman Mao Zedong and a crucial symbol of the Chinese revolution. Both scenic areas feature prominent red culture and abundant natural landscapes and have introduced new products such as red performances, red cultural creations, and red research studies, which meet the needs of this study for a diverse investigation of red tourism experience. Xibaipo is located in Hebei Province in northern China, whereas Shaoshan is in Hunan Province in central-southern China. Conducting research in these two regions allows for a balanced consideration of the north–south differences in the environmental characteristics of the scenic areas; the landscape of the research sites is shown in Figure 2.

The survey was conducted from August to October 2024 and employed a convenience sampling method. This sampling strategy, which is non-probability sampling, offers cost-effective advantages and is frequently utilized in behavioral science research (Wang et al., 2023). Four systematically trained research assistants conducted the survey at the entrance of the former site of the Central Committee of the Communist Party of China in the Xibaipo scenic area and the entrance of Mao Zedong’s former residence in the Shaoshan scenic area, respectively. The survey was administered via the WenJuanXing platform. All the participants provided informed consent before answering the questionnaire and participated entirely voluntarily. For minors, guardians’ signatures are required. The study was conducted in accordance with the Declaration of Helsinki and approved by the Biomedical Research Ethics Committee of our university (Approval No. 792).

Data quality was ensured through cleaning. Initially, we excluded 3 datasets due to formatting issues after conducting logical checks and formatting reviews. Subsequently, we removed 28 surveys with overly uniform or repetitive answers using Excel logical statements. Lastly, we discarded 23 responses with completion times more than 2.5 standard deviations below the mean (67 s), suggesting insincere responses. A total of 1,249 questionnaires were distributed, resulting in 1195 valid responses. The response rate was 95.67%. The demographic details are presented in Table 1.

**Table 1:** Demographic distribution of the sample.

Variables	Level	*n*	Percentage (*%*)
Scenic areas	Xibaipo	679	56.82
	Shaoshan	516	43.18
Gender	Male	299	25.00
	Female	896	75.00
Age	Under 18	47	3.90
	18 ~ 25	856	71.60
	26 ~ 30	17	1.40
	31 ~ 40	50	4.20
	41 ~ 50	154	12.90
	51 ~ 60	63	5.30
	Over 60	8	0.70
Education level	Junior high or below	10	0.80
	High school / secondary school	38	3.20
	Associate degree	47	3.90
	Bachelor’s degree	1,049	87.80
	Postgraduate or above	51	4.30

### 3.2 Instruments

#### 3.2.1 Red tourism experience scenario scale

The Red Tourism Experience Scenario Scale developed by Li and Zhang (2022) was utilized. This scale comprises 17 items assessing dimensions of architectural appearance, interior space, thematic exhibition, storytelling, and human–machine interaction. For instance, “The display content at the red tourism destination is well aligned with its theme,” which is rated on a 7-point scale ranging from 1 (very dissatisfied) to 7 (very satisfied). First, the item scores were standardized. A local factor-related bifactor model was constructed for calculating the reliability of the multidimensional test (Gu and Wen, 2017). Although the composite reliability (*ω*) of the total scale was 0.92, indicating high inter-item correlation and strong internal consistency, the homogeneity coefficient (*ω_h_*) was only 0.049, and the model failed to converge. This suggests that the original scale’s six-dimensional structure requires adjustment due to excessive factor correlation. Consequently, we conducted exploratory factor analysis (EFA) and confirmatory factor analysis (CFA) to reassess the scale dimensions and subsequently re-evaluated the scale’s reliability.

We performed EFA using SPSS 26.0, applying the principal component method with an eigenvalue threshold of 1 for factor extraction and employing varimax rotation. The results yielded two common factors with a combined explanatory power of 76.19%. The first factor consists of 9 items, with factor loadings ranging from 0.69 to 0.87; the second factor consists of 8 items, with factor loadings ranging from 0.70 to 0.79. Based on item content, we labeled the factors as “static landscape experience” and “dynamic interactive experience.” To further validate the scale’s structural validity, we conducted CFA. The two-factor model fit indices were as follows: *χ*^2^*/df* = 3.59, *RMSEA* = 0.08, *CFI* = 0.91, *TLI* = 0.90, and *SRMR* = 0.05^1^. Considering all the fit indices, the two-factor model fits the data acceptably, and the scale’s two-dimensional structure is considered reasonable.

The two-dimensional scale’s composite reliability (*ω*) was 0.97, reflecting high inter-item correlation and strong internal consistency. The composite reliability for each dimension was calculated using the formula for uni-dimensional composite reliability, resulting in values o*f ω*_1_ = 0.96 and *ω*_2_ = 0.95. The total scale’s homogeneity coefficient (*ω_h_*) was 0.92, with a 95% confidence interval of [0.90, 0.93], indicating that the composite score of the scale is meaningful.

#### 3.2.2 Environmental restorativeness perception scale

The Environmental Restorativeness Perception Scale revised by Guo et al. (2014) was utilized. This scale consists of 16 items covering five dimensions: coherence, novelty, fascination, escape, and compatibility. For example, for the statement “The scenery here can easily arouse my interest,” a 5 - point scale is adopted, ranging from 1 (very disagree) to 5 (very agree). Standardizing item scores led to the establishment of a bifactor model, yielding a total scale composite reliability (*ω*) of 0.98, signifying high inter-item correlation and strong internal consistency. The dimension-specific composite reliabilities were: *ω*_1_ = 0.90, *CI*_95%_ = [0.89, 0.90], *ω*_2_ = 0.86, *CI*_95%_ = [0.84, 0.87], *ω*_3_ = 0.94, *CI*_95%_ = [0.94, 0.95], *ω*_4_ = 0.92, *CI*_95%_ = [0.91, 0.92], *ω*_5_ = 0.90, *CI*_95%_ = [0.89, 0.90]. The total scale’s homogeneity coefficient (*ω_h_*) was 0.84, and *CI*_95%_ = [0.80, 0.86], suggesting that the composite score of the questionnaire is meaningful.

From a theoretical perspective, the constructs of red tourism experience and environmental restorativeness perception are complementary rather than redundant. Red tourism experience refers to visitors’ perceptions and engagement with specific “red” elements (e.g., historical awareness, emotional resonance), whereas environmental restorativeness perception focuses on how the environment helps visitors recover from mental fatigue (e.g., stress relief, attention restoration).

To test whether these constructs are empirically distinct, we modeled each as a latent variable and ran a confirmatory factor analysis (CFA) using standardized scores for each dimension. The model fit indices were χ^2^/df = 7.59, RMSEA = 0.07, CFI = 0.97, TLI = 0.95, SRMR = 0.02. Chi-square tests are known to be overly sensitive with large samples, often identifying trivial differences as significant (Bentler and Bonett, 1980). Therefore, researchers rely on alternative fit indices to evaluate model fit. In this case, all the alternative indices indicated a good fit, so the model’s overall fit was deemed acceptable.

Discriminant validity was further supported by the Fornell–Larcker criterion (Fornell and Larcker, 1981): each construct’s average variance extracted (AVE) exceeded its maximum shared variance (MSV). The red tourism experience construct had an AVE of 0.81 versus an MSV of 0.74, and the environmental restorativeness perception construct had an AVE of 0.75 versus an MSV of 0.74.

#### 3.2.3 Post-visit behavioral intention questionnaire

Numerous studies have assessed post-visit behavioral intentions using two main metrics: willingness to recommend and willingness to revisit (Barnes et al., 2016; Fu and Luo, 2023; Gallarza and Saura, 2006; Chen and Xi, 2018), typically captured through two specific questions. Consistent with prior research, the present study adopts these two indicators to measure tourists’ post-visit behavioral intentions. For instance, “If given the chance, I would travel to this place again,” rated on a 5-point scale from 1 (strongly disagree) to 5 (strongly agree). A uni-dimensional test comprising two items was conducted. After the scores of the questions were standardized, the synthetic reliability of the uni-dimensional test was 0.89, and the *CI*_95%_ = [0.88, 0.91], indicating that the internal consistency of the questionnaire was high.

#### 3.2.4 Cultural identity scale

The Simple General Cultural Identity Questionnaire, revised by Huang and Bi (2021), was used. This scale consists of nine items across two dimensions. A 7-point scale is adopted. For example, for the item “I’m full of pride in Chinese culture,” 1 represents completely disagree, and 7 represents completely agree. The total scale composite reliability is 0.96, with composite reliability for each dimension at 0.94, and the 95% confidence intervals are [0.94, 0.95] for all; the coefficient of homogeneity of the total scale is 0.82, with *CI*_95%_ = [0.88, 0.91]. This indicates a high level of internal consistency within the questionnaire, and the synthetic total score is meaningful.

### 3.3 Data processing and statistical analysis

Data organization, descriptive statistics, partial correlation analysis, hierarchical regression analysis, and exploratory factor analysis were performed using spss 26.0 software, and synthetic reliability tests, common method bias analysis, and moderated mediation model examination were performed via Mplus 8.3.

## 4 Results

### 4.1 Common method bias test

Confirmatory factor analysis was conducted on four competing models: Model 1 (M_1_), the baseline model, where all indicators load separately on their corresponding latent variables; Model 2 (M_2_), a bifactor model, which adds a global factor G as a method factor based on M_1_; Model 3 (M_3_), a combined model, merging red tourism experience, environmental restorativeness perception, and post-visit behavioral intentions into one factor, with cultural identity as another; and Model 4 (M_4_), a one-factor model, combing all indicators into a single factor.

As shown in Table 2, M_1_ demonstrates good fit across all indices and outperform M_2_, M_3_, and M_4_, suggesting strong discriminant validity among the four variables. The addition of a global factor as a method factor (i.e., M_2_) results in a decline in model fit indices. Furthermore, the Harman one-factor CFA test (i.e., M_4_) reveals poor model fit. These results suggest that the measures are not significantly affected by common method bias.

**Table 2:** Model comparison for common method bias test.

Model	χ^2^	df	AIC	BIC	RMSEA	CFI	TLI	SRMR
M_1_	4421.38	926	89749.35	90680.07	0.07	0.92	0.91	0.03
M_2_	6863.76	877	91161.72	92016.15	0.08	0.89	0.88	0.04
M_3_	14941.80	818	99157.76	99803.67	0.12	0.76	0.74	0.07
M_4_	22105.72	902	106319.68	106960.50	0.15	0.63	0.61	0.10

### 4.2 Variable correlation test

After controlling for gender, age and education level, partial correlation analysis among variables (presented in Table 3) revealed expected correlations both within and across constructs, with coefficient magnitude and direction aligning with predictions.

**Table 3:** Partial correlation coefficients of variables (*df* = 1,090).

Variables	*M*	*SD*	1	2	3	4
1. Red tourism experience	6.10	0.96	1.00			
2. Environmental restorativeness perception	4.29	0.64	0.78^**^	1.00		
3. Cultural identity	6.59	0.76	0.63^**^	0.55^***^	1.00	
4. Post-visit behavioral intentions	4.30	0.76	0.64^**^	0.81^***^	0.50^**^	1.00

^***^p < 0.001; **p < 0.01; *p < 0.05.

### 4.3 Moderated mediation model test

The latent moderated structural equation modeling (LMS) approach (Fang and Wen, 2018) was employed to test the moderated mediation model. To simplify the model, items for the latent variables were parceled using the internal consistency approach (ICA) expect for post-visit behavioral intentions. Since the latent variable of post-visit behavioral intentions contains only two items, parceling them into one indicator could lead to inflated path coefficients, model identification issues, and potentially inappropriate solutions (Wu and Wen, 2011). Following parceling, the model fit was evaluated.

#### 4.3.1 Mediation analysis

Step1: The baseline SEM without latent moderating (interaction) terms was assessed, showing a good fit: *χ*^2^ = 266.75, *df* = 59, *RMSEA* = 0.07 (*CI*_90%_ = [0.067, 0.073]), *CFI* = 0.94, *TLI* = 0.92, and *SRMR* = 0.04.

Subsequently, we conducted a supplementary analysis to assess the mediating effect of environmental restorativeness perception (Wen and Ye, 2014). The bias-corrected non-parametric percentile bootstrap method was employed to estimate the confidence intervals for the coefficients. In the first step, the direct path from red tourism experience to post-visit behavioral intentions showed good model fit: *χ*^2^ = 38.13, *df* = 10, *RMSEA* = 0.05 (*CI*_90%_ = [0.03, 0.07]), *CFI* = 0.98, *TLI* = 0.97, and *SRMR* = 0.04. After controlling for age, gender, and education level, the red tourism experience significantly predicted post-visit behavioral intentions, *β* = 0.72, *p* < 0.001; *CI*_95%_ = [0.68, 0.80], explaining 52.40% of the variance in post-visit behavioral intentions.

**Figure 3:**
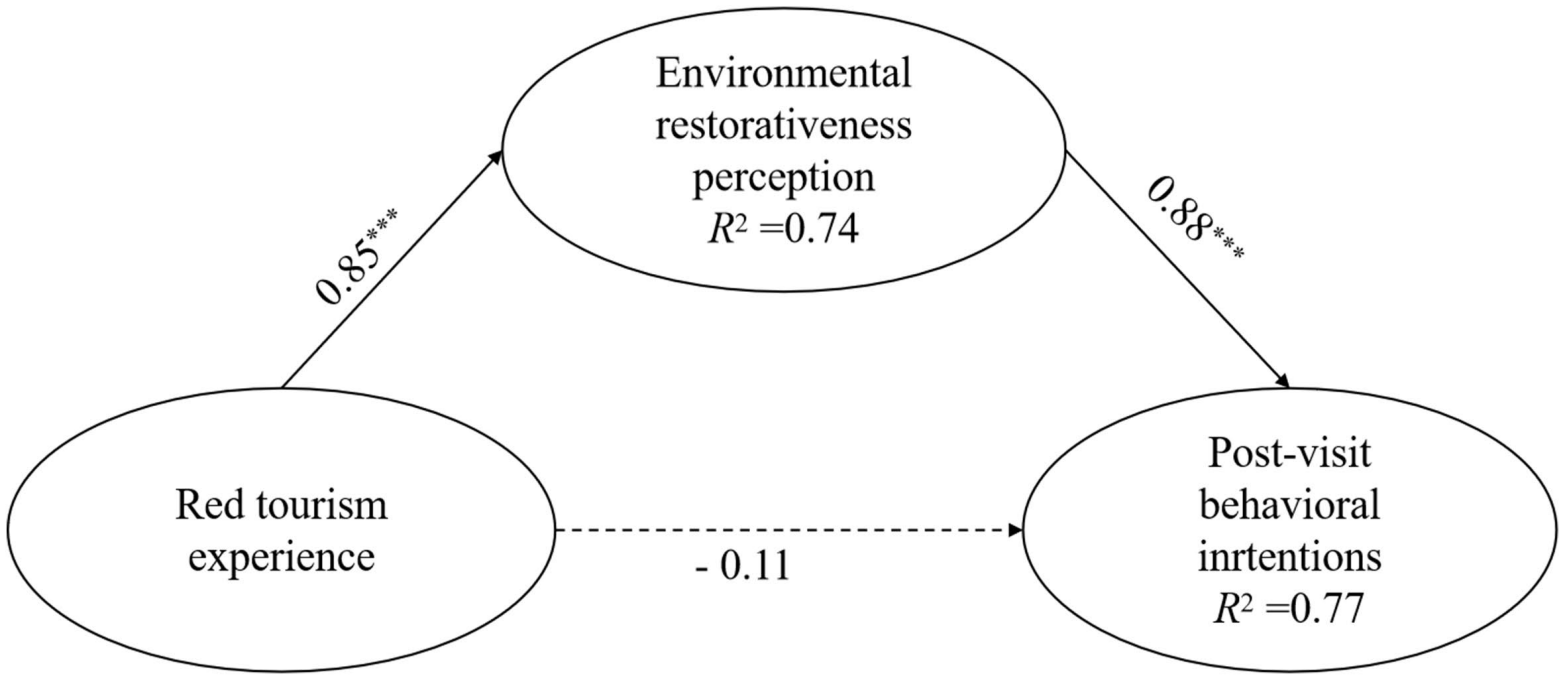
Mediating effect test results. Gender, age, and education level are included as control variables in the equations but are not displayed in the figure for the sake of simplicity. The dashed lines indicate non-significant coefficients. ***p < 0.001.

Based on the prior model, environmental restorativeness perception was incorporated as a mediating variable, and the results indicated a well-fitting model: *χ*^2^ = 422.78, *df* = 95, *RMSEA* = 0.08 (*CI*_90%_ *=* [0.08, 0.09]), *CFI* = 0.93, *TLI* = 0.90, and *SRMR* = 0.04. Environmental restorativeness perception and post-visit behavioral intentions explained 74.00 and 76.80% of the variance, respectively. Figure 3 shows that, after controlling for gender, age, and education level, red tourism experience significantly predicted environmental restorativeness perception, β = 0.85, *p* < 0.001; *CI*_95%_ = [0.81, 0.88], and environmental restorativeness perception significantly predicted post-visit behavioral intentions, β = 0.88, *p* < 0.001; *CI*_95%_ = [0.82, 0.92]. However, the direct effect of red tourism experience on post-visit behavioral intentions was not significant, *β* = −0.11, *p* > 0.05. The mediating effect accounted for 89.74% of the total effect. Therefore, environmental restorativeness perception mediated the relationship between red tourism experience and post-visit behavioral intentions, supporting Hypothesis 2.

#### 4.3.2 Moderated mediation analysis

*Step 2*: We tested the moderated mediation model using latent moderated structural equation modeling (LMS). The inclusion of interaction terms (red tourism experience × cultural identity, environmental restorativeness perception × cultural identity) improved model fit, with the Akaike information criterion (AIC) decreasing from 22421.55 to 22377.87, *δ*AIC = 43.68, and a significant log-likelihood ratio test, δ-2LL = 25.83, δ*df* = 4, *p* < 0.001, indicating that the moderated mediation SEM fits the data better than the baseline SEM does, justifying the move to Step 3.

*Step 3*: Utilizing the coefficient product method to analyze the moderated mediation effect. Cultural identity significantly moderated the relationship between red tourism experience and environmental restorativeness perception β = 0.06, *p* < 0.001, but not the paths from environmental restorativeness perception to post-visit behavioral intentions, β = −0.09, *p* > 0.05, or from red tourism experience to post-visit behavioral intentions, β = 0.08, *p* > 0.05. This indicates cultural identity moderates the first stage of the mediation pathway.

Bootstrap analysis (5,000 iterations) confirmed the indirect effect remained significant across different levels of cultural identity, while the direct effect was non-significant (Table 4). This pattern demonstrates that red tourism experience influences post-visit behavioral intentions primarily through environmental restorativeness perception rather than directly, with this mediation process moderated by cultural identity.

**Table 4:** The mediating effect of environmental restorativeness perception between red tourism experience and post-visit behavioral intentions at different levels of cultural identity.

Effects	Cultural identity	Mediation effect value	Boot SE	95% CI		*p*
				Lower	Upper	
Direct effect	*M - SD*	−0.11	0.08	−0.26	0.04	0.16
	*M*	−0.08	0.07	−0.22	0.07	0.28
	*M + SD*	−0.05	0.12	−0.28	0.18	0.67
Indirect effect	*M - SD*	0.75	0.08	0.59	0.91	0.001
	*M*	0.83	0.07	0.70	0.95	0.001
	*M + SD*	0.90	0.11	0.69	1.11	0.001

If the bootstrap confidence interval does not include 0, it indicates a significant moderated mediation effect.

**Figure 4:**
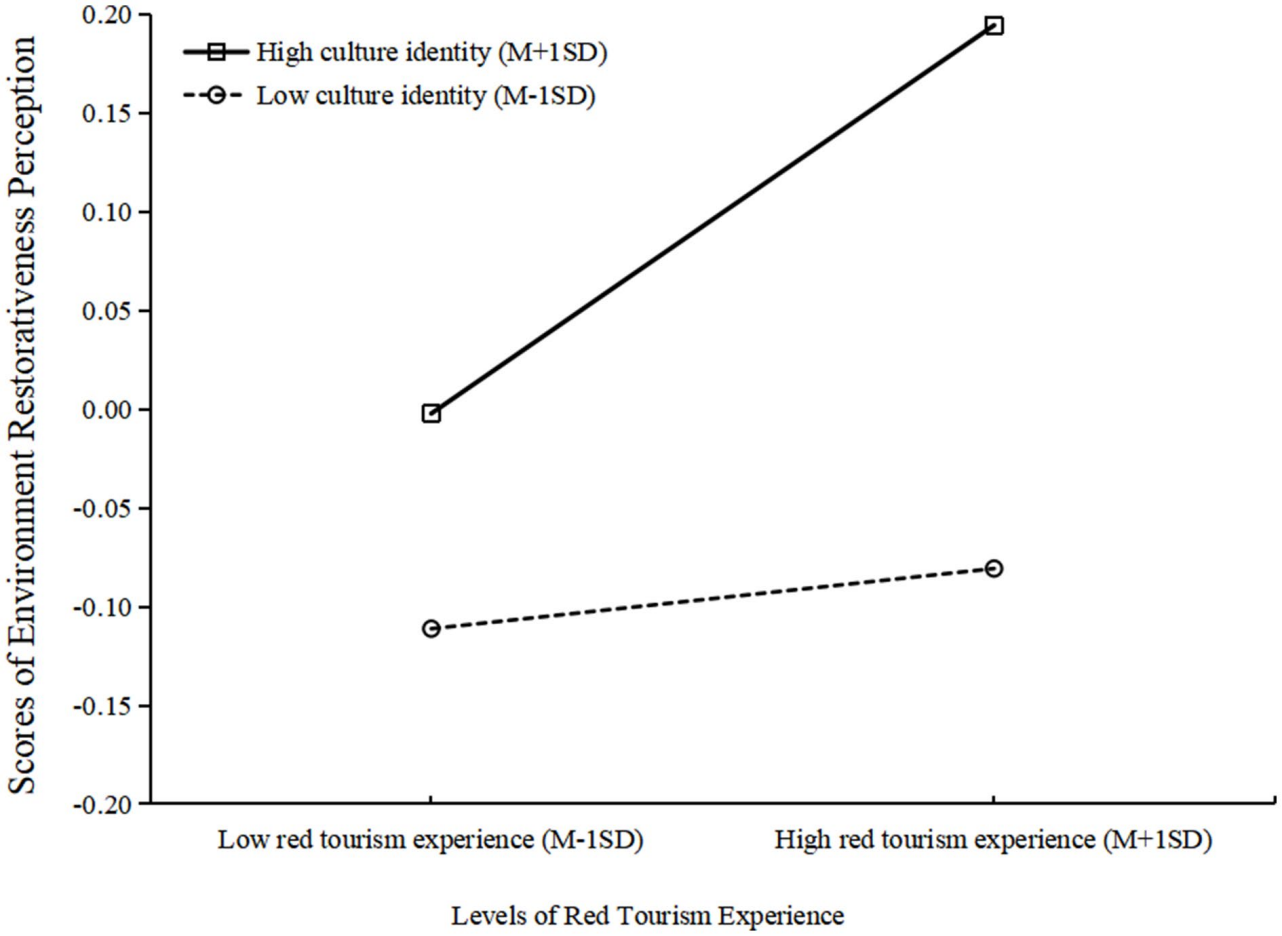
Simple slope analysis results.

Simple slope analysis (Figure 4) revealed that the positive effect of red tourism experience on environmental restorativeness perception was stronger for participants with high cultural identity (one standard deviation above the mean) compared to those with low cultural identity (one standard deviation below the mean).

**Figure 5:**
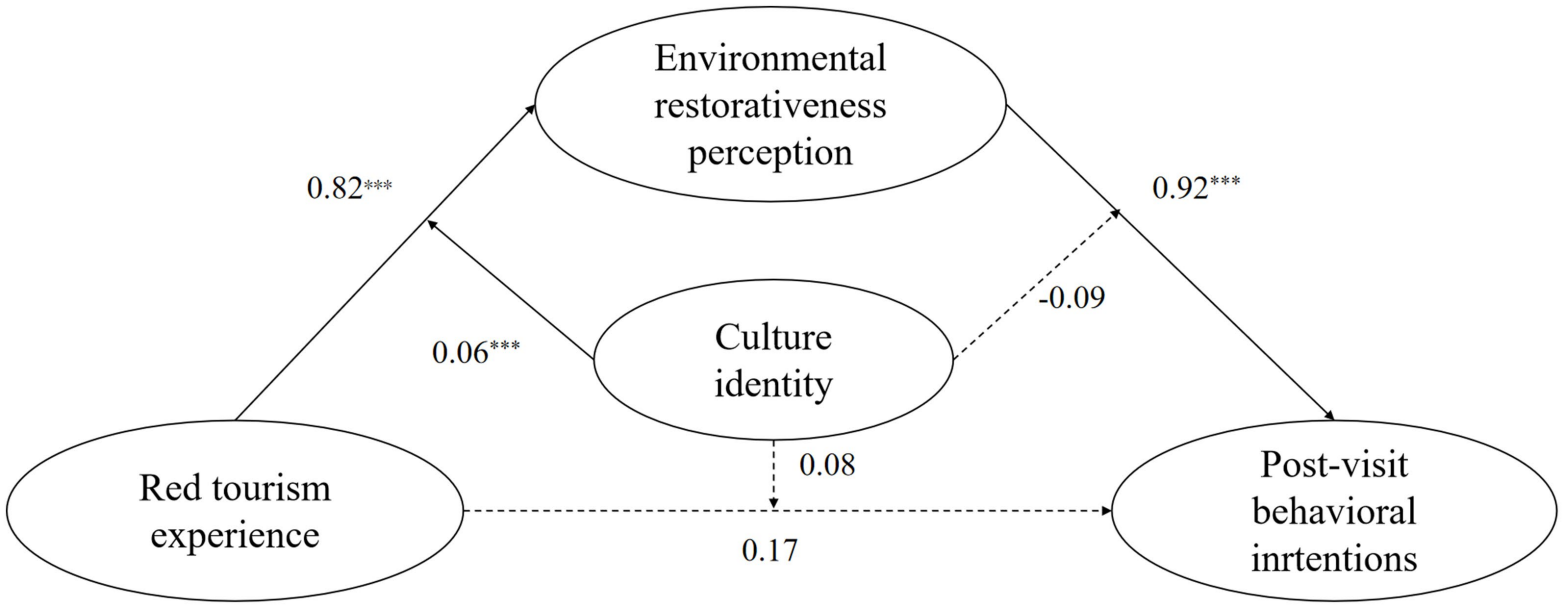
Moderated mediation model of red tourism experience, environmental restorativeness perception, and post-visit behavioral intentions with cultural identity as a moderator. ***p < 0.001.

In summary, environmental restorativeness perception mediates the relationship between red tourism experience and post-visit behavioral intentions, with cultural identity significantly moderating the first stage of this mediation pathway. Figure 5 clearly highlights these key relationships and the critical paths in our moderated mediation model.

## 5 Discussion

### 5.1 Red tourism may be categorized as a restorative environment

Identifying restorative environments and understanding their benefits is a central focus in environmental psychology. This study expands existing perspectives by exploring a broader range of restorative environment types. In this study, participants’ reported mean of environmental restorativeness perception was significantly higher than the theoretical mean, *M* = 4.29, *t*(1194) = 70.14, *p* < 0.001, *Cohen’s d* = 2.86, *effect size* (*r*) = 0.82. This large effect size indicates that visitors strongly perceived the restorative qualities of red tourism environments.

Early research on restorative environments primarily focused on natural settings and their capacity to reduce stress and mental fatigue (Berry et al., 2015; Bratman et al., 2019; Collado et al., 2017). However, more recent studies have broadened this concept beyond natural contexts (Stragà et al., 2023), increasingly acknowledging the restorative potentials of cultural heritage sites (Daelemans, 2020; Xu and Hu, 2024). For instance, heritage sites with strong cultural meaning have been found to provide restorative experiences comparable to those offered by natural settings (Scopelliti et al., 2019). Red tourism sites, characterized by historical significance, cultural narratives, and well-designed physical environments, may also possess features aligned with this expanded framework. Previous studies have demonstrated that the physical design and interpretive elements of red tourism destinations can directly strengthen their restorative potential (Zhang and Li, 2024).

Thus, red tourism environments may represent a distinct type of restorative setting, characterized by a dual-function restorative process. Which may explain why red tourism holds special appeal for tourists who seek both leisure and a deeper understanding of and emotional connection to national history and culture. On one hand, these environments provide traditional restorative benefits, including attention recovery and stress reduction, which align with foundational principles of ART. On the other hand, red tourism environments uniquely foster identity-based psychological restoration, enabling visitors to connect with collective values and construct personal meaning. This dual restorative process not only expands the current understanding of ART beyond natural environments but also highlights the importance of cultural and historical contexts as restorative resources. Moreover, this finding challenges the conventional focus on sensory-driven restoration by emphasizing the cognitive and emotional dimensions of environmental perception. Future research should explore how these identity-driven restoration processes interact with traditional restorative factors, offering a more integrative perspective on the restorative potential of political heritage environments.

### 5.2 The relationship between environmental restorativeness perception and post-visit behavioral intentions

This study confirmed that environmental restorativeness perception plays a pivotal role in shaping post-visit behavioral intentions, corroborating prior findings (Chen et al., 2024; Lin and Yang, 2024; Chen and Xi, 2018; Huang et al., 2022). Nevertheless, there is an ongoing debate about the predictive role of each dimension of environmental restorativeness perception (Chen and Xi, 2018; Huang et al., 2022). To comprehensively discuss this issue, this study augmented the analysis with hierarchical multiple regression. The results showed that all the regression equations were significant. After controlling for variables such as gender, age and education level, the “coherence” and “fascination” dimensions of environmental restorativeness perception significantly predicted post-visit behavioral intentions (see Table 5). The non-significant results for these other dimensions suggest that not all aspects of environmental restorativeness are equally important in the red tourism context. This finding diverges from Chen’s research results (Chen and Xi, 2018), in which “coherence” was not regarded as a predictive factor for post-visit behavioral intentions. This discrepancy likely arises from variations in the types of environments studied.

**Table 5:** Hierarchical regression analysis of environmental restorativeness perception on post-visit behavioral intentions.

Outcome variable	Step	Predictor variable	First step	Second step	*F*	*R* ^2^	*δR* ^2^
			*β*	*β*			
Y1	First	Gender	0.22^***^	0.22^***^	12.27^***^	0.03	0.03
		Age	−0.01	0.01			
		Education level	0.22^***^	0.14^**^			
	Second	Coherence	/	0.25^***^	78.20^***^	0.35	0.34
		Novelty	/	−0.01			
		Fascination	/	0.33^***^			
		Escape	/	−0.02			
		Compatibility	/	0.05			
Y2	First	Gender	0.22^***^	0.22^***^	12.26^***^	0.03	0.03
		Age	0.03	0.04			
		Education level	0.23^***^	0.15^***^			
	Second	Coherence	/	0.27^***^	67.24^***^	0.31	0.31
		Novelty	/	0.03			
		Fascination	/	0.29^***^			
		Escape	/	−0.06			
		Compatibility	/	0.04			

The standardized scores of each dimension of environmental restorativeness perception were input as predictor variables in the second layer, and standardized scores of the two dimensions of post-visit behavioral intentions were analyzed as outcome variables. ^***^p < 0.001; **p < 0.01; *p < 0.05.

This differential impact of restorativeness dimensions underscores a key theoretical insight: the context-specificity of restorative mechanisms. While ART posits universal dimensions of restorative environments, our findings indicate that their relative importance can shift depending on the environmental context. Within red tourism settings, coherence emerges as particularly important. This result expands current theoretical frameworks by suggesting that the psychological processes underlying restoration differ between ideologically charged environments and natural or generic built environments.

In the context of red tourism, “coherence” typically refers to how well tourism projects, services, and products align with the red cultural theme and how consistently and accurately they convey the values of red culture. As sites that preserve revolutionary history and culture, it is essential that all displayed content, explanatory texts, and activities accurately reflect historical events and the spirit of red culture to ensure educational credibility and seriousness. Furthermore, coherence extends to multiple aspects of the tourist journey, including services, facilities, and activities, all of which must align with the red tourism theme to enhance the visitor experience. Thus, coherence is more critical for post-visit behavioral intentions in red tourism compared to other dimensions. This finding challenges the prevailing assumption that fascination is universally the most important driver of restorative experiences. Instead, it suggests that in environments with strong cultural and historical narratives, the clarity and coherence of these narratives may play a greater role in facilitating restoration and influencing subsequent behavior. These results not only refine our understanding of restorativeness constructs but also point to a need for further theoretical exploration into how contextual factors, such as cultural and ideological consistency, redefine what constitutes a “restorative” environment in heritage tourism.

### 5.3 The mediating role of environmental restorativeness perception between red tourism experience and post-visit behavioral intentions

This study found that environmental restorativeness perception mediates the relationship between red tourism experience and post-visit behavioral intentions, as the direct predictive effect of red tourism experience on behavioral intentions became non-significant. This indicates that positive tourism experiences significantly enhanced visitors’ environmental restorativeness perception, which in turn strongly predicted post-visit behavioral intentions. In other words, when visitors perceive the environment as restorative, they are more likely to exhibit positive behaviors, such as recommending the destination or revisiting it. Although we initially hypothesized both direct and indirect effects, our data supported only an indirect pathway via the perception. This finding suggests that simply offering positive experiences is not sufficient to foster favorable post-visit behavioral intentions; rather, the red tourism experience should specifically enhance environmental restorativeness perception.

The results support Sun and Chen’s perspectives (Chen and Chen, 2010; Sun et al., 2019), indicating that the relationship between red tourism experience and post-visit behavioral intentions is mediated by environmental restorativeness perception. On one hand, tourism experiences from sensory stimuli provide significant psychological and physiological restorative benefits to visitors (Zhang and Li, 2024). These experiences lead to internal perceptual assessments, evoke personal emotions, and encourage reflections on both reality and the self (Zhou G. et al., 2023; Zhou H. et al., 2023). Namely, a positive red tourism experience boosts environmental restorativeness perception. On the other hand, as visitors experience restorative benefits such as stress relief, emotional enhancement, and attention restoration (Zhang and Li, 2024), these perceptions shape their evaluations of the destination and ultimately influence positive post-visit behaviors (Huang et al., 2022).

This mediation model bridges previously disconnected theoretical frameworks in environmental psychology and tourism studies, specifically linking ART with the theory of planned behavior. By positioning restorativeness as the key psychological mechanism connecting experience to behavioral intention, our findings suggest that the success of ideological tourism environments may depend depends not on direct persuasion but on their ability to generate psychologically restorative experiences. This represents a novel theoretical perspective on how built environments with strong cultural-political narratives influence visitor behavior.

This result supports the SOR theory. Specifically, tourists form internal states of energy, positive emotions, and deep reflection through external stimuli such as the appearance of scenic spots, internal spaces, thematic exhibitions, storytelling, interpersonal interactions, and participation in activities, which ultimately lead to their decision-making behaviors. However, our current model relies on a single mediating variable and does not account for other potential mediators such as emotional or cognitive factors. Future research should validate additional mediating pathways and broaden the framework by incorporating diverse mediators, as well as exploring how these mechanisms operate across different cultural and environmental contexts. Expanding sample diversity and representativeness, as well as examining various types of tourism settings, will further clarify how tourism experiences influence post-visit behavioral intentions.

### 5.4 Moderating role of cultural identity in the mediating effect

Studies show that cultural identity strongly affects positive post-visit behavioral intentions and is pivotal in shaping these decisions (Usunier, 2006; White et al., 2022; Yang et al., 2021). Our structural equation model analysis reveals that cultural identity moderates only the first half of the mediation path. Specifically, tourists with high cultural identity exhibited a markedly greater increase in environmental restorativeness perception as their red tourism experiences deepened. Contrary to our expectations, cultural identity neither moderated the latter segment of the mediating pathway (from environmental restorativeness perception to post-visit behavioral intentions) nor the direct effect of red tourism experience on post-visit behavioral intentions. This pattern highlights a complex temporal dynamic in how cultural and environmental factors interact, suggesting that the impact of cultural identity diminishes once restorative qualities are perceived, ultimately warranting further exploration into the sequential nature of these interactions.

These findings reveal an important theoretical nuance: cultural identity primarily influences how experiences are processed into restorative perceptions rather than how these perceptions translate into behavioral intentions. This challenges the conventional view of cultural identity as a uniform moderator throughout the tourism experience—behavior pathway. Instead, our results suggest a more intricate mechanism wherein cultural identity acts as a perceptual filter during the experience phase, enhancing the interpretation of restorative qualities, but becomes less relevant during the decision-making phase. In other words, once restorative qualities are perceived, they exert a consistent influence on post-visit behavior, regardless of cultural identification levels.

These nuanced insights into the role of cultural identity are consistent with person-environment fit theory, which suggests that the stronger the alignment between an individual’s cultural values and the destination’s ideological narrative, the more pronounced the restorative effect. As a significant form of cultural tourism, red tourism emphasizes cultural depth and educational value. During red tourism, visitors acquire knowledge, interact with red culture, and undergo emotional experiences. Strong cultural identification embedded in red tourism indicates a robust person-environment fit, enriching tourists’ overall experiences. Consequently, tourists benefit from attentional resource restoration, increased positive emotions, and deep reflection, all of which contribute to positive post-visit behavioral intentions.

This selective moderation effect provides empirical support for an integrated theoretical framework that combines person-environment fit theory with ART. It suggests that cultural-ideological alignment primarily enhances the perception of restorative qualities rather than directly influencing behavioral outcomes. These findings contribute to resolving theoretical tensions between universalist approaches in environmental psychology and cultural-relativist perspectives. By showing both universal (restorativeness-behavior link) and culturally specific (experience-restorativeness link) mechanisms operate within the same behavioral pathway, these results broaden our understanding of how restorative and cultural factors interact in heritage tourism contexts.

### 5.5 Red tourism as a restorative pathway to transformative experience

Previous studies indicate that engaging with natural environments and cultural activities can trigger transformative experiences (Godovykh, 2024). Building on this foundation, this study reveals that red tourism, as a restorative environment imbued with ideological meaning, not only facilitates the recovery of tourists’ cognitive and emotional resources, but also promotes spiritual elevation through the reinforcement of cultural identity, thereby generating transformative experiences centered on well-being enhancement.

First, unlike prior studies that emphasize disruptive or challenging encounters (e.g., witnessing poverty) as transformation triggers (Pung et al., 2020), red tourism engages visitors through a positive and gentle pathway, as conceptualized by the four characteristics of restorative environments outlined in Attention Restoration Theory. These characteristics operate as follows: fascination captures involuntary attention, reducing cognitive fatigue; being away enables detachment from daily stressors, decreasing anxiety; compatibility enhances person-environment fit and satisfaction; coherence creates immersive flow experiences through unified narratives. These attributes collectively enable cognitive restoration and emotional activation, subsequently fostering self-growth and psychological resilience through historical reflection and cultural connection. This process of cognitive restoration and emotional activation essentially represents the transformation of ordinary travel experiences into profound well-being enhancement.

Second, beyond environmental factors, cultural identity serves as the psychological foundation for meaningful engagement with red tourism environments and acts as a catalyst for restorative perception. Tourists with stronger cultural identification perceive heroic narratives and historical artifacts as embodiments of collective memory, evoking positive emotions such as belonging and purpose, thus fostering well-being through collective identity reinforcement. This indicates that well-being enhancement varies according to individual cultural backgrounds and value orientations rather than following a uniform pattern.

In summary, our findings provide empirical insights into how ideologically themed tourism fosters psychological well-being, enriching transformative experience theory applications in culture-specific contexts.

## 6 Practical implications

This study offers insights into managing and innovatively developing red tourism. First, the tourism experience is vital to the tourism economy (Zhou et al., 2022), and enriching the red tourism experience through diverse methods is crucial. By deeply exploring local unique resources and integrating the red gene, we can promote the integration of red tourism with ecotourism and rural tourism, achieving resource sharing and complementary advantages. Second, integrating cultural heritage tourism with technology is a growing trend (Zhang et al., 2023). Technological means should be actively utilized to enhance visitor experiences. For example, serious games (Zhang J. et al., 2024; Zhang R. et al., 2024), virtual reality technology, and multidimensional presentation techniques can be employed to develop unique technological tourism products. By combining red historical culture with contemporary culture, we can create technologically advanced and culturally rich tourism routes, facilitating in-depth interaction among visitors, the environment, and others. Moreover, it is crucial to fully utilize the sensory elements of sight, sound, touch, smell, and taste to construct a comprehensive and multilayered sensory experience for visitors, making each red tourism trip an unforgettable spiritual and cultural exploration.

Secondly, enhancing the restorative qualities of the red tourism environment is crucial. The restorative environment is highly associated with tourists’ psychological and behavioral reactions (Backman et al., 2023). Although many studies have investigated the restorative function of the natural environment, the restorative aspect of the cultural environment has been relatively overlooked (Chang and Li, 2022). Our study reveals that the red tourism environment is restorative and that tourists’ environmental restorativeness perceptions positively predict their post-visit behavioral intentions. Notably, the dimensions of coherence and fascination play significant predictive roles. Therefore, enhancing the restorative aspects of red tourism, particularly its coherence and fascination, should be prioritized in development and management. In terms of coherence, it is essential to ensure the coordination of the tourism destination environment so that all the elements fit well with the overall setting. For instance, emphasize the organic integration of red tourist sites with surrounding natural landscapes, folk cultures, etc., and closely link tourist projects, interpretations, activities, and services with the local red cultural theme throughout the entire tourism process. In terms of fascination, the “novelty sense” can be emphasized. For example, innovative forms can be created through projects such as red script murder and role-playing, making the red spirit more dynamic. Highlight the “context sense” by using scene simulation and 3D technology to build immersive exhibitions, restoring historical scenes and evoking deeper emotions. Highlight the “substitution sense” by integrating immersive interactions, enabling tourists to participate in plot deductions and become “witnesses” of historical events, thus achieving a profound connection between the experience of historical content and the red cultural theme.

Third, integrating culture with environment is essential for the prosperity and sustainability of the tourism industry. Red tourism should concentrate on creating a strong cultural memory and emotional atmosphere by deeply exploring revolutionary history, heroic deeds, and spiritual values. For instance, cultural-themed activities such as making Red Army straw sandals can provide hands-on experiences that deepen tourists’ identification with red culture. Moreover, organizing agricultural experience activities can allow tourists to better understand the hardships of the revolutionary era while learning about local history and culture. Such activities strengthen their connection to both revolutionary and national cultural identities. In this way, red tourism serves as a process of inheriting red culture (Huang and Zhu, 2008). Enriching the tourism environment with diverse cultural activities not only boosts its restorative qualities but also forges a profound connection between historical narratives, the essence of red culture, and the surrounding environment. Together, these efforts establish a “culture empowerment—environment enhancement” dual strategy that promotes positive post-visit behavioral intentions.

This dual-dimensional approach also aligns with the ideological-landscape psychological mechanism identified in this study. Wherein visitors’ environmental perceptions are strengthened through identification with specific cultural narratives. By reinforcing visitors’ environmental perceptions through stronger identification with specific cultural narratives, site managers can optimize both the quality of the visitor experience and the resulting behavioral outcomes. By simultaneously activating visitors’ cultural identification and environmental immersion through well-coordinated cultural programming and intentional environmental design, this strategy moves beyond traditional dichotomies that separate cultural/educational and environmental/aesthetic management. Instead, it demonstrates how these dimensions can work synergistically to enhance visitor experiences and improve outcomes when properly integrated.

## 7 Limitations and future research directions

This study has certain limitations that present opportunities for future research. First, our measurement of post-visit behavioral intentions focused primarily on recommendation and revisit dimensions; future studies could incorporate objective indicators such as social media engagement metrics and actual revisitation rates to enhance research depth.

Second, our model did not account for potential alternative pathways or additional variables that might influence the relationship between red tourism experiences and post-visit behavioral intentions. Future research could explore mediating factors beyond environmental restorativeness perception and examine multi-mediator models (Fu and Luo, 2023; Wang et al., 2023), providing more comprehensive insights into tourist behavior mechanisms within red tourism contexts.

Third, the complex moderating role of cultural identity in tourism experiences and decision-making processes warrants further investigation. Future studies could incorporate additional potential moderators such as patriotism and historical interest, which might explain additional variance in the model (Fu and Luo, 2023; Ferguson et al., 2017; Yang et al., 2021). Additionally, comparative studies examining how cultural identity functions across different forms of heritage tourism could yield valuable insights. Finally, as our sample was limited to visitors at Chinese red tourism sites, the generalizability of findings may be constrained. Extending this research to other forms of political heritage tourism globally would help determine whether the identified restorative benefits and behavioral mechanisms are unique to red tourism or applicable to broader contexts of cultural and ideological tourism experiences (Scopelliti et al., 2019; Tresidder and Deakin, 2019; Zhang et al., 2023).

## 8 Conclusion

Red tourism may represent a unique form of restorative environment that integrates both landscape and cultural identity dimensions. Specifically, environmental features such as “coherence” and “fascination” significantly predicted tourists’ post-visit behavioral intentions. Environmental restorativeness perception served as a mediator between red tourism experience and post-visit behavioral intentions, with no significant direct effect, indicating that the restorative experience is a key psychological mechanism driving post-visit behavioral intentions. Moreover, the first stage of this mediation pathway was significantly moderated by cultural identification, indicating that in ideologically charged landscapes, tourists’ identification with specific cultural narratives enhances their perception of the environment. These findings support a dual-path strategy of “cultural empowerment and environmental enhancement,” whereby the synergy between cultural connection and environmental design fosters both cultural identity and psychological restoration, ultimately promoting positive post-visit behavioral intentions. Future research should explore additional moderating and mediating variables, particularly the interplay between cultural and environmental characteristics, to refine the theoretical model and increase its practical relevance.

## Data Availability

The raw data supporting the conclusions of this article will be made available by the authors, without undue reservation.

## Footnotes

^1^The evaluation of structural equation model fit was conducted using multiple established fit indices as recommended by Hu and Bentler (1999). Acceptable model fit was determined using the following criteria: Chi-square/df ratio (χ^2^/df) < 3.0; Comparative Fit Index (CFI) ≥ 0.90 and Tucker-Lewis Index (TLI) ≥ 0.90; Root Mean Square Error of Approximation (RMSEA) < 0.08 with 90% confidence interval upper bound < 0.10; and Standardized Root Mean Square Residual (SRMR) < 0.08. These thresholds represent conventional standards in behavioral and social science research, balancing model parsimony with explanatory power.
